# Improved outcomes for severely injured children in designated pediatric trauma centers in the Netherlands

**DOI:** 10.1007/s00068-025-02916-5

**Published:** 2025-07-14

**Authors:** Sem A. M. Jansen, Manouk Backes, Dominique B. Buck, Anneliese Nusmeier, Lucas Timmermans, Stefan W. A. M. van Zutphen, Michael J.R. Edwards, Erik Hermans, Stijn D. Nelen

**Affiliations:** 1 Department of Trauma Surgery, Radboud University Medical Center, Nijmegen, the Netherlands; 2 Department of Pediatric Surgery, Radboud University Medical Center, Nijmegen, the Netherlands; 3 Pediatric Intensive Care, Amalia Children’s Hospital, Radboud University Medical Center, Nijmegen, The Netherlands; 4 Department of Surgery, Elisabeth TweeSteden Hospital, Tilburg, The Netherlands

**Keywords:** Pediatric trauma, Trauma centers, Mortality, Glasgow outcome scale, Treatment outcome

## Abstract

**Purpose:**

Pediatric trauma centers (PTCs) have been associated with lower mortality rates and increased use of non-operative management. While not formally designated, six trauma centers in the Netherlands meet PTC criteria and are referred to as designated pediatric trauma centers (dPTCs). This study aimed to evaluate the impact of treatment at dPTCs versus adult trauma centers (ATCs) on outcomes in severely injured pediatric patients in the Netherlands.

**Methods:**

Data were obtained from the Dutch National Trauma Registry for patients aged ≤ 16 years with an Injury Severity Score (ISS) ≥ 16, admitted between January 1, 2015, and December 31, 2022. Multivariable logistic regression was performed to assess the impact of treatment at a dPTC on in-hospital mortality and Glasgow Outcome Scale (GOS) scores.

**Results:**

In total, 2,378 patients were included: 63% were treated in dPTCs, 17% in ATC-I, and 20% in ATC-II/III. Mortality rates were 13.1% in dPTCs, 12.6% in ATC-I, and 2.1% in ATC-II/III (*p* < 0.001). For children under 12 years of age, treatment at a dPTC was independently associated with a lower risk of in-hospital mortality compared to ATC-I (odds ratio [OR] 1.99, *p* = 0.017). dPTC treatment was also associated with more favorable GOS outcomes compared to ATC-I (OR 0.68, *p* = 0.022) and ATC-II/III (OR 0.34, *p* < 0.001).

**Conclusion:**

Treatment at dPTCs is associated with a reduced risk of mortality for patients under 12 years of age and improved functional neurological outcomes. These findings support the further centralization of pediatric trauma care in the Netherlands.

**Supplementary Information:**

The online version contains supplementary material available at 10.1007/s00068-025-02916-5.

## Background

In the Netherlands, an annual average of 320 multitrauma patients (ISS ≥ 16) aged 16 years or younger were admidded to emergency departments, the majority of whom were between 12 and 16 years old. The in-hospital mortality rate within this pediatric trauma population was 8.2%, most often due to road traffic accidents [1].

It is crucial to emphasize that managing pediatric trauma cases presents unique challenges due to the substantial differences in anatomy and pathophysiology in children compared to adults. These inequalities significantly influence the interpretations of physical findings and further examinations [2–4]. It is therefore essential that healthcare providers adapt their clinical approach accordingly. Therefore, alongside the regular adult trauma centers (ATCs), pediatric trauma centers (PTCs) have been established, primarly outside of Europe, to provide the highest quality of care for the pediatric population. Studies from the United States (US) have shown that pediatric trauma patients with an Injury Severity Score ≥ 16, treated at PTCs, were more frequently managed non-operatively, particularly in cases involving head, liver, and spleen injuries. Additionally, a decrease in overall mortality rates was observed [4].

PTCs have become a standard component of trauma care in countries such as the US, United Kingdom, Switzerland, and Australia [2, 4–6]. In contrast, the Netherlands has no officially recognized PTCs. However, six Dutch trauma centers meet the verification criteria set by the American College of Surgeons (ACS), including 24/7 availability of pediatric surgical specialists, a dedicated Pediatric Intensive Care Unit (PICU), access to pediatric subspecialties, child life services, and pediatric-specific trauma protocols [7, 8]. In this study, these Dutch centers are referred to as designated pediatric trauma centers (dPTCs). Table 1 summarizes the key ACS criteria for Pediatric Trauma Centers.

**Table 1:** Key criteria for pediatric trauma center designation according to the American college of surgeons (ACS) [7–9]

Domain	Key ACS requirements
24/7 pediatric specialist access	Pediatric surgical and anesthesiology expertise must be continuously available.
PICU availability	On-site Pediatric Intensive Care Unit staffed 24/7 by pediatric intensivists.
Subspecialty services	Access to pediatric neurosurgery, orthopedics, urology, and other specialties.
Emergency readiness	Pediatric resuscitation protocols and size-appropriate equipment must be in place.
Imaging and diagnostics	Pediatric-specific imaging protocols and timely radiologist interpretation required.
Child life and family support	Certified child life specialists and family-centered support services must be available.
Rehabilitation services	Access to pediatric physical, occupational, and speech therapy for post-trauma recovery
Protocols and quality programs	Institutions must maintain pediatric trauma protocols and participate in quality programs.

To date, no research has explored the impact of dPTCs compared to ATCs in the Netherlands. Therefore, the primary aim of this study was to assess the effect of treatment at a dPTC versus an ATC on in-hospital mortality among pediatric trauma patients. Secondary outcomes included the Glasgow Outcome Scale (GOS) at discharge and the total length of hospital stay.

## Method

### Data collection

A retrospective cohort study was performed to identify severely injured pediatric trauma patients who were admitted to trauma centers in the Netherlands during the period from January 1 st, 2015, to December 31 st, 2022. The data was acquired from the Dutch National Trauma Registry (DNTR), a prospective database that comprehensively records all patients who sustained injuries and were admitted to a hospital through the Emergency Department (ED) [10]. The study focused on severely injured pediatric patients, therefore only patients with an injury severity score (ISS) ≥ 16 and aged 16 and younger were included.

Patient characteristics extracted from the DNTR were age, gender, and comorbidities as defined by American Society of Anesthesiologists (ASA) score [9]. Injury characteristics included trauma mechanism (blunt or penetrating injuries), ISS and Abbreviated Injury Scale (AIS) [11, 12]. Prehospital data included utilization of the Helicopter Emergency Medical Service (HEMS) [13]. Hospital characteristics encompassed both the patient’s primary admitting hospital and, if applicable, the final hospital after transfer. This was categorized as either a dPTC or one of the ATC levels, including ATC-I and ATC-II/III. dPTCs were defined according to the criteria established by the American College of Surgeons (ACS), as outlined in Table 1 [4, 7, 14]. If patients were initially admitted to an ATC and transferred to a dPTC within 48 h, they were considered to have been treated at a dPTC in the analyses. For those patients who were transferred to a dPTC, patient demographics and primary and secondary outcomes were compared between those transferred from ATC-I to dPTC and those transferred from ATC-II/III to dPTC.

The primary outcome measure of the study was in-hospital mortality. The secondary outcome measures included the Glasgow Outcome Scale (GOS), administered at discharge, and the total length of hospital stay (LOS) in days, encompassing both the general ward and the intensive care unit (ICU) [15].

### Statistical analysis

Descriptive statistics were used to characterize the three study groups, severely injured pediatric trauma patients treated at a dPTC versus those treated at an ATC-I versus those treated at an ATC-II/-III.

Primary and secondary outcome measures were analyzed and reported for all study groups.

Continuous variables were described using either means and standard deviations or medians with interquartile range (IQR, 25th–75th percentile) when the data followed a non-normal distribution. Categorical variables were described using frequencies and percentages. Univariate analysis included independent t-tests and one-way ANOVA for parametric data and Mann–Whitney U and Kruskal–Wallis tests for nonparametric data. Fisher’s exact tests and Chi-square tests were used for categorical data. These univariate analyses were conducted in two phases: first, a comparison was made between dPTC and ATC-I, denoted as ‘P value between dPTC and ATC-I.’ Additionally, analyses were performed comparing dPTC, ATC-I, and ATC-II/III as three groups, referred to as ‘P value overall’.

Finally, explanatory variables with a p-value < 0.10 in the initial univariate analysis were included in a multivariable binary logistic regression model and a multivariable ordinal regression model to assess the impact of treatment at a dPTC on in-hospital mortality and on the GOS, respectively. The multivariable analyses were reiterated for the age cohort under 12 and under 8 years of age. Odds ratios (ORs), 95% confidence intervals (CIs), and p-values were reported. A p-value of < 0.05 was considered significant. All statistical analyses were carried out using SPSS software (version 29.0; IBM SPSS Statistics, Armonk, NY).

## Results

**Fig. 1:**
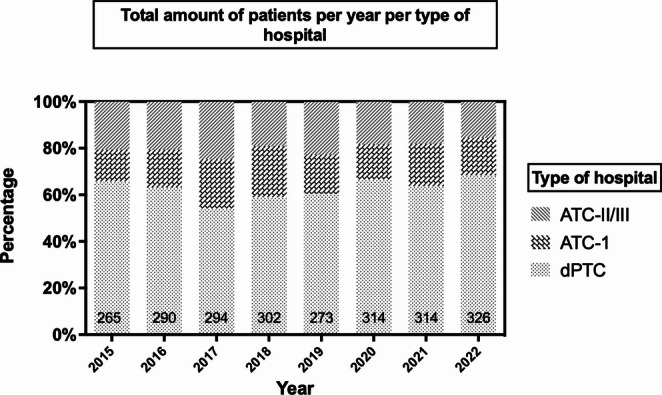
Distribution of severely injured pediatric trauma patients per year per type of hospital (in percentages)

A total of 2,378 patients met the inclusion criteria, of whom 1,491 (63%) were treated in a dPTC, 413 (17%) in an ATC-I, and 474 (20%) in an ATC-II/III. The distribution of the pediatric patients among the hospitals over the years is shown in Fig. 1.

The analysis of baseline characteristics revealed that patients in a dPTC had a significantly lower age (median 10, IQR 5–14, *p* < 0.001). The proportion of male patients was similar for all centers, with 62–65% being male. There was a significant difference in ISS score between dPTC (ISS 22, IQR 17–26) and ATC-I (ISS 21, IQR 17–26) vs. ATC-II/III (ISS 17, IQR 16–25) (overall *p* < 0.001), but there was no significant difference between dPTC vs. ATC-I (*p* = 1.000). Significantly more children presented with head injuries in dPTCs (61.6%), followed by 55.0% in ATC-I and 48.1% in ATC-II/III (overall *p* < 0.001). For abdominal injuries, significantly fewer children were treated in dPTCs (20.7%), followed by 21.5% in ATC-I and 33.5% in ATC-II/III (overall *p* < 0.001). Patients treated in dPTCs had a significantly higher incidence of HEMS involvement (38.1% in dPTC, 22.6% in ATC-I, and 3.8% in ATC-II/III, overall *p* < 0.001) (Table 2).

**Table 2:** Comparison of baseline characteristics of severely injured pediatric trauma patients treated at a dPTC versus those treated at an ATC-I or ATC-II/III

Characteristics		dPTC *N* = 1491	ATC-I *N* = 413	*P* value between dPTC and ATC-I	ATC-II/III *N* = 474	*P* value overall
		Median (IQR)				
Age		10 (5–14)	12 (5–15)	0.000	11 (5–15)	< 0.001
ISS score		22 (17–26)	21 (17–26)	1.000	17 (16–25)	< 0.001
		Numbers (%)				
Gender	Female	542 (36.4)	145 (35.1)	0.642	181 (38.2)	0.625
ASA classification	I	1168 (88.1)	380 (92.2)	0.019	374 (91.0)	0.030
	II	136 (10.3)	31 (7.5)	0.100	31 (7.5)	0.105
	III/IV/V	22 (1.7)	1 (0.2)	0.028	6 (1.5)	0.091
Mechanism of injury	Blunt	1441 (97.0)	397 (97.1)	0.967	445 (98.5)	0.250
AIS score ≥ 3	Head	919 (61.6)	227 (55.0)	0.014	228 (48.1)	< 0.001
	Face	21 (1.4)	11 (2.7)	0.079	5 (1.1)	0.117
	Neck	13 (0.9)	4 (1.0)	0.773	1 (0.2)	0.303
	Thorax	329 (22.1)	97 (23.5)	0.540	53 (11.2)	< 0.001
	Abdomen	309 (20.7)	89 (21.5)	0.715	159 (33.5)	< 0.001
	Spine	76 (5.1)	19 (4.6)	0.682	14 (3.0)	0.151
	Upper extremity	19 (1.3)	2 (0.5)	0.284	2 (0.4)	0.139
	Lower extremity	156 (10.5)	54 (13.1)	0.134	29 (6.1)	0.002
	Extern	151 (10.1)	54 (13.1)	0.087	37 (7.8)	0.035
Prehospital HEMS involvement	Yes	561 (38.1)	93 (22.6)	< 0.001	17 (3.8)	< 0.001

Univariate analyses revealed that patients treated in an ATC-II/III exhibited significantly lower mortality rates (2.1%) compared to 13.1% in dPTC and 12.6% in ATC-I (*p* < 0.001).

For the children treated in the dPTC, the smallest percentage entered a vegetative state (0.7% in dPTC, 5.1% in ATC-I, and 1.1% in ATC-II/III, overall *p* < 0.001), and the majority were discharged with a moderate disability (40.0% in dPTC, 33.4% in ATC-I, and 32.7% in ATC-II/III, overall *p* = 0.002). Patients treated in dPTCs had significantly longer lengths of stay on the general ward and in the intensive care unit, with medians of 7 days (IQR 4–13) and 2 days (IQR 1–5), respectively (*p* < 0.001) (Table 3).

**Table 3:** Primary and secondary outcomes measures of severely injured pediatric trauma patients treated at a dPTC versus those treated at an ATC-I or ATC-II/III

		dPTC *N* = 1491	ATC-I *N* = 413	*P*-value between dPTC and ATC-I	ATC-II/III *N* = 474	*P*-value overall
		Numbers (%)				
Hospital mortality		196 (13.1)	52 (12.6)	0.780	10 (2.1)	< 0.001
GOS	Vegetative state	11 (0.7)	21 (5.1)	< 0.001	5 (1.1)	< 0.001
	Severe disability	219 (14.7)	57 (13.8)	0.348	122 (25.7)	< 0.001
	Moderate disability	596 (40.0)	138 (33.4)	< 0.001	155 (32.7)	0.002
	Good recovery	293 (19.7)	120 (29.1)	< 0.001	68 (14.3)	< 0.001
	Missing	191 (12.8)	25 (6.1)		115 (24.3)	
		Median (IQR)				
Hospital length of stay in days		7 (4–13)	4 (1–8)	0.000	1 (1–2)	< 0.001
ICU length of stay in days		2 (1–5)	1 (0–2)	0.000	0 (0–0)	< 0.001

For children under 12 years of age, patient characteristics and primary and secondary outcome measurements were also analyzed (Supplementary information (SI) Table 1 and SI Table 2).

Multivariable binary logistic regression analysis for children under 16 years of age did not reveal treatment in a dPTC to be an independent predictor of a lower mortality risk (Table 4).

**Table 4:** Multivariable binary logistic regression analyses for hospital mortality, of severely injured pediatric trauma patients treated at a dPTC versus those treated at an ATC-I or ATC-II/III

Variables		Odds ratio (95% CI)	*P*-value
Age		0.937 (0.909 to 0.967)	< 0.001
ASA	ASA I	0.284 (0.098 to 0.823)	0.020
	ASA II	0.349 (0.107 to 1.141)	0.081
	ASA III/IV/V	Reference	
ISS		1.108 (1.091 to 1.125)	< 0.001
Prehospital HEMS involvement		4.586 (3.183 to 6.608)	< 0.001
Type of hospital:	dPTC	Reference	
	ATC-I	1.284 (0.837 to 1.971)	0.253
	ATC-II/III	0.521 (0.236 to 1.151)	0.107

However, analysis for children under 12 years of age did show this association, compared to treatment in an ATC-I (OR 1.99, 95% confidence interval (CI) 1.13–3.50, *p* = 0.017) (Table 5).

**Table 5:** Multivariable logistic regression analyses for hospital mortality, of severely injured pediatric trauma patients < 12 years old, treated at a dPTC versus those treated at an ATC-I or ATC-II/III

Variables		Odds ratio (95% CI)	*P*-value
Age		0.853 (0.769 to 0.914)	< 0.001
ASA	ASA 1	0.191 (0.048 to 0.760)	0.019
	ASA II	0.338 (0.072 to 1.599)	0.171
	ASA III/IV/V	Reference	
ISS		1.122 (1.096 to 1.150)	< 0.001
Prehospital HEMS involvement		5.351 (3.187 to 8.985)	< 0.001
Type of hospital	dPTC	Reference	
	ATC-I	1.989 (1.130 to 3.502)	0.017
	ATC-II/III	0.361 (0.095 to 1.367)	0.134

Multivariable ordinal regression analysis for children under 16 years of age revealed dPTC to be an independent predictor of more favorable GOS compared to treatment in ATC-II/III (OR 0.37, 95% CI 0.30–0.47, *p* < 0.001) (Table 6). In the multivariable analysis for children under 12 years of age only, dPTC was also found to be an independent predictor for more favorable GOS compared to both ATC-I (OR 0.68, 95% CI 0.49–0.95, *p* = 0.022) and ATC-II/III (OR 0.34, 95% CI 0.25–0.47, *p* < 0.001) (Table 7).

**Table 6:** Multivariable ordinal logistic regression analyses for GOS, of severely injured pediatric trauma patients treated at a dPTC versus those treated at an ATC-I or ATC-II/III

Variables		Odds ratio (95% CI)	*P*-value
Age		1.012 (0.996 to 1.029)	0.139
ASA	ASA I	1.214 (0.536 to 2.750)	0.641
	ASA II	1.152 (0.751 to 1.769)	0.516
	ASA III/IV/V	Reference	
ISS		0.910 (0.900 to 0.921)	< 0.001
Prehospital HEMS involvement		0.322 (0.262 to 0.394)	< 0.001
Type of hospital:	dPTC	Reference	
	ATC-I	0.924 (0.736 to 1.160)	0.496
	ATC-II/III	0.373 (0.295 to 0.473)	< 0.001

**Table 7:** Multivariable ordinal logistic regression analyses for variables GOS, of severely injured pediatric trauma patients < 12 years old treated at a dPTC versus those treated at an ATC-I or ATC-II/III

Variables		Odds ratio (95% CI)	*P*-value
Age		1.024 (0.992 to 1.057)	0.149
ASA	ASA I	1.118 (0.432 to 2.895)	0.819
	ASA II	1.143 (0.685 to 1.907)	0.609
	ASA III/IV/V	Reference	
ISS		0.910 (0.895 to 0.924)	< 0.001
Prehospital HEMS involvement		0.290 (0.220 to 0.382)	< 0.001
Type of hospital:	dPTC	Reference	
	ATC-I	0.681 (0.489 to 0.946)	0.022
	ATC-II/III	0.339 (0.247 to 0.465)	< 0.001

For both the binary regression and the ordinal regression, sub-analyses were also performed for children under 8 years of age. These analyses showed similar results to the analyses for children under 12 years of age.

The LOS data for both the general ward and ICU were skewed and could not be corrected, making a multivariable analysis unfeasible.

Some patients in the dPTC group initially received treatment at a different type of center before they were transferred to the dPTC within 48 h. Specifically, 59 patients were transferred from ATC-I, and 379 from ATC-II/III. Among the patients transferred from ATC-I, the mean ISS score was 24 (IQR 17–30), which was significantly higher than the mean ISS score of 17 (IQR 16–25) (*p* < 0.001) observed in patients transferred from ATC-II/III. The mortality rate in the ATC-I transfer group was also higher, with a rate of 11.9%, compared to 1.3% in the ATC-II/III transfer group (*p* < 0.001) (SI Table 3 and SI Table 4).

## Discussion

This retrospective cohort study is the first in the Netherlands to provide a comprehensive insight into how dPTCs enhance pediatric trauma care. Surprisingly, only 63% of severely injured children were treated in dPTCs, with even one in five children receiving treatment in ATC-II/III, particularly those with abdominal injuries. However, treatment in dPTCs was independently associated with more favorable GOS scores, and was also independently associated with a lower mortality risk in children under 12 years of age.

Trauma centers are designated at various levels (i.e., Level 1–3) to ensure appropriate patient allocation based on injury severity and resource availability [3, 14]. In the Netherlands, dPTCs functionally operate as Level 1 centers with pediatric specialization, whereas ATC centers often have more limited resources and less experience in managing severe pediatric trauma [14].

The improved outcomes observed in dPTCs—particularly the lower mortality in children under 12 years of age and better functional recovery—are likely attributable to the structural and organizational advantages defined by the ACS. dPTCs offer continuous access to pediatric surgical and critical care specialists, operate with pediatric-specific protocols, and have dedicated resources such as PICUs, child life services, and pediatric subspecialties including neurosurgery, anesthesiology, and radiology. These elements enable rapid and tailored interventions for severely injured children. In contrast, ATCs often lack these pediatric-specific infrastructure, which may require inter-hospital transfers of critically injured children, leading to potential delays in definitive care.

In addition, literature shows that high-volume centers with pediatric trauma expertise generally achieve lower mortality rates and improved outcomes due to increased provider familiarity and institutional experience [16].

The mortality rate of children treated in a dPTC was 13.1%. This mortality rate is comparable to mortality rates for severely injured pediatric patients in other countries, such as 11.9% in the US, 14% in Spain, 13.4% in Germany, or even lower compared to 26,2% in Switzerland and 30% in Finland [4, 17–19]. This study did not reveal treatment in a dPTC to be an independent predictor for a lower mortality risk when compared to treatment in ATC-II/III, possibly due to the higher incidence of neurotrauma in dPTCs, which is associated with high mortality [20, 21].

Regarding functional outcomes and LOS, a study in the US also investigated both outcomes among severely injured pediatric patients treated at dPTCs. Similar to our findings, the study also revealed that pediatric patients treated in a dPTC have favorable functional outcomes. But in contrast to our study, it reported a shorter LOS for children treated in a dPTC. The longer LOS in our study may be due to the high incidence of neurotrauma in dPTCs, which can lead to impaired consciousness and behavior and may delay discharge due to challenges in arranging follow-up care or rehabilitation placement [21, 22].

This is the first study in the Netherlands to compare outcomes between pediatric trauma patients treated in dPTCs versus those treated in ATCs in a nationwide study. However, our study was limited by several factors. Firstly, we encountered a high number of missing values in the GOS. Secondly, this study was unable to determine whether differences in outcomes among transferred patients were due to the transfer itself or the treatment received at the dPTC, leaving the influence of transferred patients on outcomes unclear. The GOS in dPTCs may have been negatively influenced by the lower GOS in children transferred from ATC-I, while children transferred from ATC-II/III might have positively influenced the dPTC results due to lower mortality and better GOS. Additionally, our study lacked in-hospital data on factors contributing to care provision in dPTCs, such as the frequency of surgical interventions or imaging [4]. These limitations underscore the need for further research to comprehensively understand and implement the factors contributing to improved outcomes in dPTCs.

Overall, our findings demonstrate that dPTCs in the Netherlands may provide better outcomes for severely injured pediatric patients, particularly for children under 12 years of age, with lower odds of mortality and higher odds of improved neurological outcomes compared to ATCs. These results support further centralization of pediatric trauma care in the Netherlands to improve the quality of care for this patient group. Transitioning a dPTC into a formal PTC, however, requires the integration of comprehensive pediatric services, such as child protection experts, behavioral health specialists, social work support, educational resources, and rehabilitation facilities. Future efforts should focus on optimizing these services to ensure multidisciplinary care for severely injured children and to address the needs of their families.

## Electronic supplementary material

Below is the link to the electronic supplementary material.

Supplementary Material 1

## Data Availability

The data used in this study are derived from the Dutch National Trauma Registry (DNTR) and are not publicly available. However, they may be made available by the corresponding author upon reasonable request and with explicit permission from the DNTR
