# Supplementary material for: Improved outcomes for severely injured children in designated pediatric trauma centers in the Netherlands
Source: Eur J Trauma Emerg Surg. 2025 Jul 14;51(1):253. doi: 10.1007/s00068-025-02916-5 (PMC12259715; doi:10.1007/s00068-025-02916-5)
Supplement: Supplementary file 1 — Supplementary Material 1 [file 68_2025_2916_MOESM1_ESM.docx]

*Supplementary information*

*SI Table 1***:** Comparison of baseline characteristics of severely injured pediatric trauma patients <12 years old, treated at a dPTC versus those treated at an ATC-I or ATC-II/III.

| **Characteristics** | | **dPTC**  ***N= 871*** | ***ATC I***  ***N= 192*** | ***P value between dPTC and ATC-I*** | **ATC II/ III *N= 262*** | ***P value overall*** |
| --- | --- | --- | --- | --- | --- | --- |
|  | | **Median (IQR)** | | | | |
| **Age** | | 5 (2-8) | 5 (2-8) | 0.059 | 6 (3-9) | <0.001 |
| **ISS score** | | 21 (17-26) | 21 (17-25) | 0.400 | 17 (16-25) | <0.001 |
|  | | **Numbers (%)** | | | | |
| **Gender** | **Female** | 319 (36.6) | 71 (37.0) | 0.934 | 100 (38.2) | 0.902 |
| **ASA classification** | **I** | 694 (89.0) | 180 (93.8) | 0.060 | 215 (94.3) | 0.015 |
|  | **II** | 72 (9.2) | 11 (5.7) | 0.149 | 10 (4.4) | 0.029 |
|  | **III/IV/V** | 14 (1.8) | 1 (0.5%) | 0.327 | 3 (1.3) | 0.415 |
| **Mechanism of injury** | **Blunt** | 849 (97.9) | 185 (98.4) | 1.000 | 245 (98.4) | 0.840 |
| **AIS score ≥ 3** | **Head** | 554 (63.6) | 107 (55.7) | 0.048 | 149 (56.9) | 0.037 |
|  | **Face** | 10 (1.1) | 2 (1.0) | 1.000 | 2 (0.8) | 0.867 |
|  | **Neck** | 8 (0.9) | 1 (0.5) | 1.000 | 1 (0.4) | 0.625 |
|  | **Thorax** | 141 (16.2) | 35 (18.2) | 0.520 | 18 (6.9) | <0.001 |
|  | **Abdomen** | 163 (18.7) | 33 (17.2) | 0.681 | 73 (27.9) | 0.003 |
|  | **Spine** | 25 (2.9) | 4 (2.1) | 0.806 | 5 (1.9) | 0.620 |
|  | **Upper extremity** | 7 (0.8) | 0 (0.0) | 0.363 | 1 (0.4) | 0.375 |
|  | **Lower extremity** | 67 (7.7) | 16 (8.3) | 0.767 | 9 (3.4) | 0.042 |
|  | **Extern** | 109 (12.5) | 43 (22.4) | <0.001 | 26 (9.9) | <0.001 |
| **Prehospital**  **HEMS**  **involvement** | **Yes** | 322 (37.5) | 50 (26.3) | 0.003 | 8 (3.2) | <0.001 |

*SI Table 2***:** Primary and secondary outcomes measures of severely injured pediatric trauma patients <12 years old, treated at a dPTC versus those treated at an ATC-I or ATC-II/III.

|  | | **dPTC**  ***N= 871*** | **ATC- I**  ***N= 192*** | ***P-value between dPTC and ATC-I*** | **ATC II/III**  ***N=262*** | ***P-value overall*** |
| --- | --- | --- | --- | --- | --- | --- |
|  | | **Numbers (%)** | | | |  |
| **Hospital mortality** | | 110 (12.6) | 31 (16.2) | 0.195 | 5 (1.9) | <0.001 |
|  | **Vegetative state**  **Severe disability**  **Moderate disability**  **Good recovery**  **Missing** | 8 (1.1)  106 (14.1)  343 (45.5)  193 (25.6)  117 (13.4) | 12 (6.7)  25 (13.9)  55 (30.6)  57 (31.7)  12 (6.3) | <0.001  1.000  <0.001  0.111 | 3 (1.5)  72 (35.6)  82 (40.6)  41 (20.3)  60 (22.9) | <0.001  <0.001  0.001  0.040 |
|  | | **Median (IQR)** | | | |  |
| **Hospital length of stay in days** | | 6 (3-12) | 2 (1-6) | <0.001 | 1 (1-2) | <0.001 |
| **ICU length of stay in days** | | 2 (1-4) | 0 (0-2) | <0.001 | 0 (0-0) | <0.001 |

*SI Table 3***:** Comparison of baseline characteristics of severely injured pediatric patients transferred from an ATC-I or an ATC-II/III to a dPTC.

| **Characteristics** | | **Referred from ATC-I**  ***N= 59*** | **Referred from ATC-II/III**  ***N= 379*** | ***P-value*** |
| --- | --- | --- | --- | --- |
|  | | **Median (IQR)** | | |
| **Age** | | 10 (5-13) | 9 (4-13) | 0.868 |
| **ISS score** | | 24 (17-30) | 17 (16-25) | <0.001 |
|  | | **Numbers (%)** | | |
| **Gender** | **Female** | 22 (37.3) | 136 (35.9) | 0.884 |
| **ASA classification** | **I** | 52 (88.1) | 303 (79.9) | 1.000 |
|  | **II** | 6 (10.2) | 35 (9.2) | 1.000 |
|  | **III/ IV/V** | 1 (1.7) | 7 (1.8) | 0.088 |
| **Mechanism of injury** | **Blunt** | 58 (98.3) | 377 (99.5) | 0.252 |
| **AIS score ≥3** | **Head** | 46 (78.0) | 221 (58.3) | 0.004 |
|  | **Face** | 1 (1.7) | 0 (0) | 0.135 |
|  | **Neck** | 2 (3.4) | 0 (0) | 0.018 |
|  | **Thorax** | 13 (22.0) | 26 (6.9) | <0.001 |
|  | **Abdomen** | 7 (11.9) | 137 (36.1) | <0.001 |
|  | **Spine** | 4 (6.8) | 7 (1.8) | 0.047 |
|  | **Upper extremity** | 0 (0) | 1 (0.3) | 1.000 |
|  | **Lower extremity** | 3 (5.1) | 19 (5.0) | 1.000 |
|  | **Extern** | 3 (5.1) | 4 (1.1) | 0.055 |
| **Prehospital HEMS involvement** | | 10 (16.9) | 17 (4.5) | <0.001 |

*SI Table 4***:** Primary and secondary outcome measures of severely injured pediatric patients transferred from an ATC-I or an ATC-II/III to a dPTC.

|  | | **Referred from ATC-I**  ***N= 59*** | **Referred from ATC-II/III**  ***N= 379*** | ***P-value*** |
| --- | --- | --- | --- | --- |
|  | | **Numbers (%)** | | |
| **Hospital mortality** | | 7 (11.9) | 5 (1.3) | < 0.001 |
| **GOS** | **Vegetative state**  **Severe disability**  **Moderate disability**  **Good recovery**  **Missing** | 0 (0)  25 (42.4)  20 (33.9)  6 (10.2)  1 (1.7) | 1 (0.3)  32 (8.4)  172 (45.4)  119 (31.4)  51 (13.5) | 1.000  <0.001  0.015  <0.001 |
|  | | **Median (IQR)** | | |
| **Hospital length of stay in days** | | 7 (3-19.5) | 6 (3.5-9) | 0.140 |
| **ICU length of stay in days** | | 4 (2-6) | 2 (0-3) | <0.001 |
